# Fine Mapping of the Gene Controlling the Fruit Skin Hairiness of *Prunus persica* and Its Uses for MAS in Progenies

**DOI:** 10.3390/plants10071433

**Published:** 2021-07-14

**Authors:** Zhenhua Lu, Lei Pan, Bin Wei, Liang Niu, Guochao Cui, Luwei Wang, Wenfang Zeng, Zhiqiang Wang

**Affiliations:** National Peach and Grape Improvement Center, Key Laboratory of Fruit Breeding Technology of Ministry of Agriculture, Zhengzhou Fruit Research Institute, Chinese Academy of Agricultural Sciences, Zhengzhou 450009, China; luzhenhua@caas.cn (Z.L.); panlei@caas.cn (L.P.); weibinbin2016@163.com (B.W.); niuliang@caas.cn (L.N.); cuiguochao@caas.cn (G.C.); 15839552412@163.com (L.W.); zengwenfang@caas.cn (W.Z.)

**Keywords:** peach, fine mapping, fruit skin hairiness

## Abstract

The fruit skin pubescence of *Prunus persica* is an economically important characteristic and comprises the classification criteria. The mapping and identification of a complete linkage marker to the fruit skin trichome trait locus of peach fruit are critical for the molecular marker-assisted selection for peach/nectarine. In this study, the BC_1_ population was constructed from the parents “Zhongyou No. 4”, the recurrent parent, and “Baihuashanbitao”, the non-recurrent parent. Based on the 38 BC_1_ individuals’ phenotypes and their genotyping using next-generation sequencing, the *G* (Glabrous skin) locus of the gene was first identified between 14.099 and 16.721 Mb on chromosome 5. Using other individuals of this population, the gene was fine-mapped in the range of 481 kb with SNP markers. Based on the resequencing data of other cultivars (lines), the candidate SNP in the gene Prupe.5G196400 was obtained. Subsequently, the SNP marker was designed and applied to natural and hybrid peach populations. Via genotyping analysis, we confirmed co-segregation between the peach/nectarine phenotype, which was used in the identification of peach or nectarine with 100% accuracy.

## 1. Introduction

Peach (*Prunus persica* L. Batsch) is one of the most important deciduous fruit trees in China, accounting for 826,325 ha of cultivation area in 2018. It is in high demand because of its flavor and high amount of nutrients [1]. As one of its important phenotypic characteristics, skin hairiness or non-hairiness (fuzziness) is considered standard for peach classification [2,3]. Before 1990, hairy peach varieties were popular and widely cultivated in China [4]. In recent years, the production and commercialization of nectarine varieties in China has increased, and some new varieties have been released. Nectarine, characterized by the absence of fruit trichomes, have a bright appearance and low allergenicity [5,6], creating high demand for them. Peaches and nectarines are native to China, with a long cultivation history [7]. However, the production and commercialization of nectarine varieties in China only started recently. Since the 1980s, breeders in China have used pollen from the USA and Italy to cross the hybrids “Jingyu” and “Qiuyu”, which has resulted in the release of several varieties [8].

To locate and identify the genes controlling the fruit skin hairiness of *Prunus persica*, markers with which to finely map the peach/nectarine locus based on the hybrid population have been developed. The RAPD marker OPZ-03 was first screened [9], and the genetic distance between OPZ-03 and the G/g locus was 23.4 cM. Since then, many Chinese researchers have been developing several markers, such as OPP20-2200, SCP20-2258, UDP96-018, and CPSCT030, which have genetic distances to the *G/g* locus as short as 4.5 cM. However, the genotyping results for these markers in natural peach varieties are not satisfactory, and the related candidate genes have not been screened. The genetic characteristics of nectarine were first reported by Blake et al. in 1932; they are controlled by the *G* locus and are recessive to normal peach [10]. The *G* (nectarine) locus was first mapped at the proximal end (bottom) of linkage group 5 [11] and is located at the distal part of linkage group 5, spanning a region from 15,126,681 to 16,315,341 (1.189 Mb), with reference to the peach genome [2,12,13,14]. An indel in candidate gene *PpeMYB25*, controlling the peaches’ skin hair, has been identified. The insertion of the LTR transcription factor in the third exon of *PpeMYB25* in the *MYB* gene family led to the appearance of a recessive hairless phenotype [2]. Recently, Cao et al. [15] associated the trait at position 17,576,893 Mb on Chromosome 3, using GWAS, and found the candidate gene ppa010316m, accounting for 80% of the explained variation.

The cultivation of peaches, as a perennial fruit tree, using traditional breeding methods is impeded by the long juvenile stage, significantly restricting the development of the peach industry [16,17]. In this study, we used the BC_1_ population from the peach cultivar “Zhongyou No. 4” to finely map the gene controlling skin hairiness (*G*) and to find molecular markers linked to the trait, with the aim of establishing a reliable SNP maker for peach/nectarine identification. Our results provide technical support for parental selection and early identification of hybrid progenies in peach/nectarine breeding.

## 2. Results

### 2.1. Genotyping with NGS

**Figure 1:**
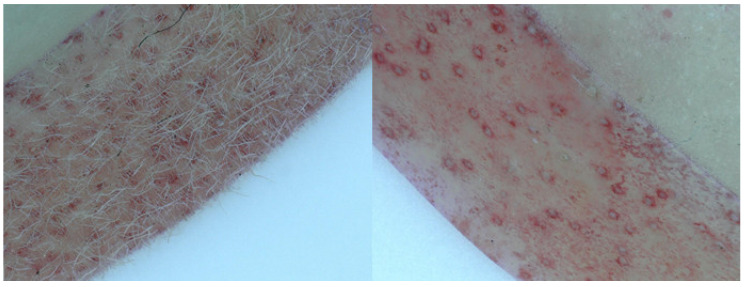
Comparing the differences between hairy fruit skin (**left**) and non-hairy skin (**right**) observed using a zoom-stereo microscope at a magnification of 30 ×.

**Figure 2:**
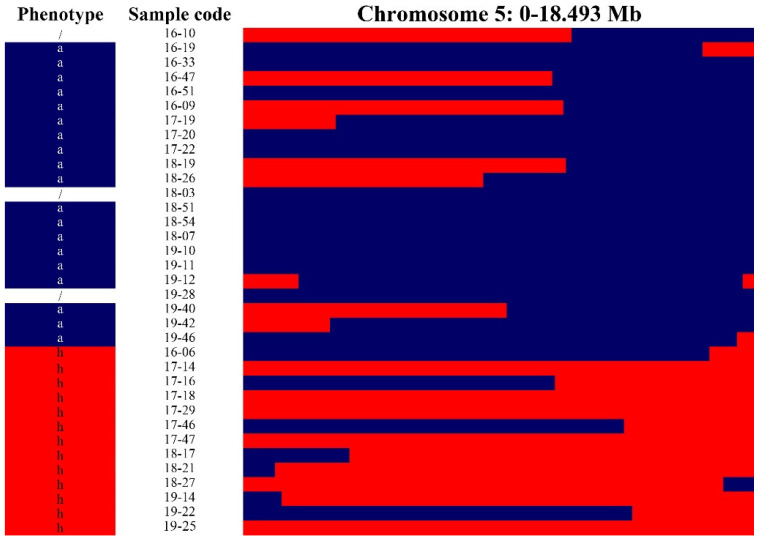
Mapping of the locus based on the genotyping and phenotype of 38 BC_1_ introgression individuals and two parents. Red indicates the introgression fragment from “Baihuahsanbitao”, blue indicates the fragments from “Zhongyou No. 4”. The letter “a” indicates homozygous nectarines; “h” indicates heterozygous peaches.

**Figure 3:**
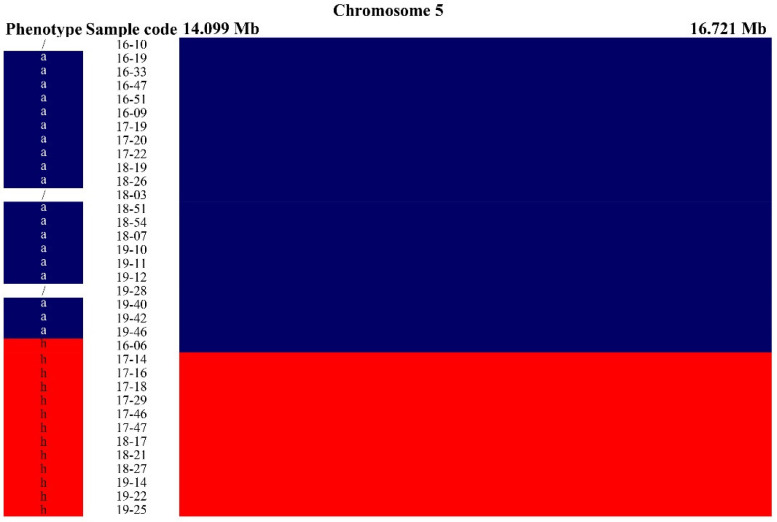
Complete linkage region with fruit skin hairiness/non-hairiness. Red indicates the introgression fragment from “Baihuahsanbitao”, blue indicates the fragments from “Zhongyou No. 4”. The letter “a” indicates homozygous nectarines; “h” indicates heterozygous peaches.

The peach or nectarine characteristics in the BC_1_ population were distinguished in the field based on fruit skin appearance. For further observation, we used a stereomicroscope to observe the differences in the fruit skin between peaches and nectarines. At 30× magnification, the peach cultivars showed white filiform growth, which was absent in the nectarine cultivars. In the progenies, the phenotype was segregated into hairy and non-hairy (Figure 1). The phenotypes of nectarines were labeled “a” and those of peaches were labeled “h”. The parents and 38 BC_1_ populations were sequenced with 70× and 10× depths, respectively. The peach genome (version 2.0) was used to select the reference genome for genotyping 38 BC_1_ individuals, with scanning of the whole peach genome (chromosomes 1–8). We found a region with the same genotypes and phenotypes on chromosome 5 (Figure 2; Supplementary Data S1), with a complete linkage region with peaches/nectarines (Figure 3; Supplementary Data S2).

### 2.2. Mapping and Fine-Mapping of Hairiness/Non-Hairiness Based on Introgression Lines

**Figure 4:**
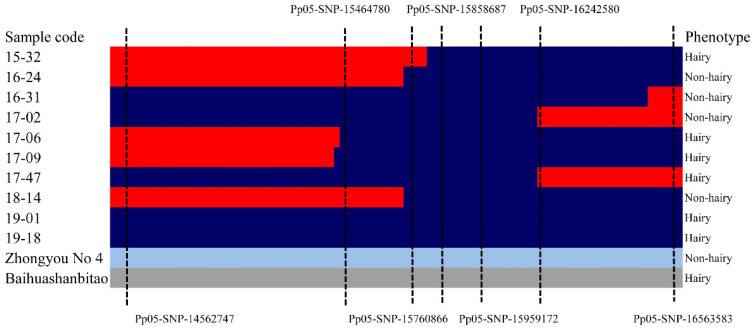
Genotype and phenotype of ten recombinants and two parents using 129 BC_1_ Individuals for fine-mapping.

To acquire closely linked SNP makers, seven SNP primer pairs (Table 1) were designed in the range of Pp05 14.099–16.721 Mb, based on the deep sequencing data for two parents. We used the 129 individuals from the Baihuashanbitao × (Zhongyou No. 4 and Baihuashanbitao) BC_1_ segregating population to finely map the locus. Both SNPs that were homozygous in “Zhongyou No. 4” and heterozygous in “Baihuashanbitao” were selected based on the parental resequencing data. Based on the SNP markers using Sanger sequencing, we identified 10 recombinants on both sides of the desired locus. Of these recombinants, the target region was narrowed down to the 481 kb region on chromosome 5, which was delimited to an interval between SNP-15760886 and SNP-16242580 by using these recombinants in the BC_1_ population. We obtained two SNP markers that were completely linked to the peach/nectarine phenotypes, namely, Pp05-SNP-15858687 and Pp05-SNP-15959172 (Figure 4).

### 2.3. Candidate Gene Confirmation Based on the Resequencing Data of Two Parents and Other Cultivars (Lines)

The reference peach genome database (version 2.0) contained 85 known transcripts within 481 kb of the fine-localization region. To acquire the candidate gene, we analyzed the correlation between the genotypes and phenotypes of the parents and 38 BC_1_ individuals. An SNP mutation site (G-A) within the Prupe.5G196400 CDS region was identified based on resequencing data for two parents (Supplementary Figure S1). To validate the candidate gene, the SNP variation in ORF of Prupe.5G196400 for 38 BC_1_ population individuals and for other 16 cultivars (lines) was further verified (Table 2). The results show that the phenotype and genotype matching rate of the site in both parents and the BC_1_ population was 100% (Supplementary Figure S2). In the finely mapped region, 86 genes were contained (Supplementary Data S3) and we determined Prupe.5G196400, with four exons and three introns, encoding a protein of unknown function, as a confident molecular marker to distinguish the peach/nectarine traits.

### 2.4. MAS of Hairiness/Non-Hairiness in Peach Breeding

**Figure 5:**
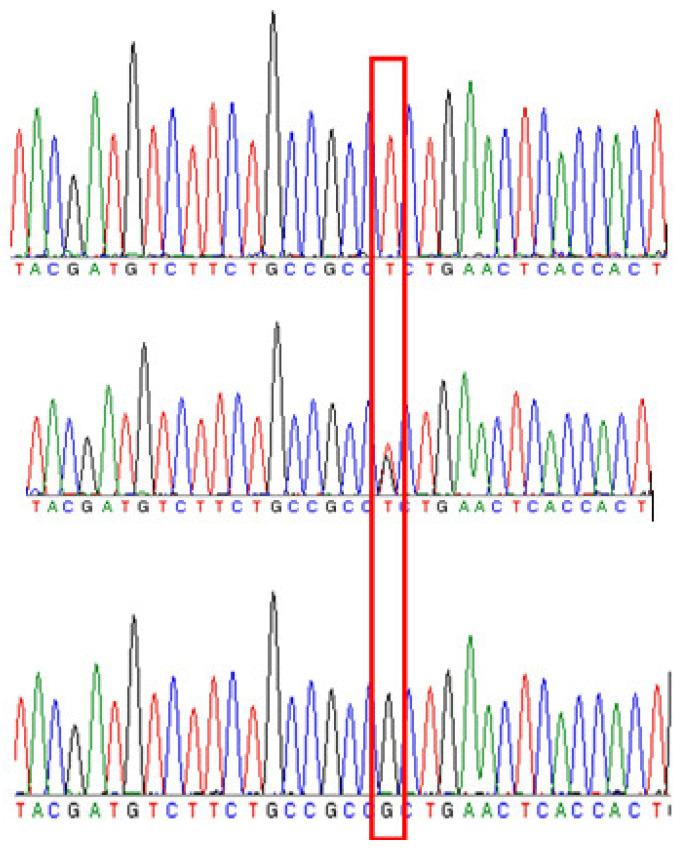
Three types of SNPs (T/T, T/G, and G/G) linked to peaches’ skin hairiness/non-hairiness in 122 cultivars (lines), based on Sanger sequencing results.

To confirm the feasibility of the identification of peach and nectarine varieties with the SNP maker in the coding region of Prupe.5G196400, 122 cultivars or lines were tested. Based on the Sanger sequencing results, there were three genotypes of SNPs (G/G, T/G, and T/T) in 122 cultivars (lines) (Figure 5). Among them, G/G and T/G represented homozygous and heterozygous genotypes of peach, respectively, and the T/T represented the homozygous genotypes of nectarines. The genotypes of SNP-Prupe.5G196400 in the 122 peach germplasm resources are shown in Supplementary Data S4. The coincidence rate of phenotypes and genotypes was 100%; thus, SNP-Prupe.5G196400 was completely linked to the *G* locus and can be used in molecular marker-assisted selection of target varieties.

In addition, three populations were used to identify the phenotype of the offspring, using the SNP marker. The result indicates that the SNP (T/T, G/T, or G/G) markers can distinguish the phenotypes of the individuals with 100% accuracy (Table 3).

## 3. Discussion

In China, peaches have been cultivated for more than 3000 years [18]. Because of their long juvenile period, it is important to determine tightly linked molecular markers to predict some of the economic traits, especially fruit traits, with the aim of establishing molecular marker-assisted selection in breeding [19]. With the development of next-generation sequencing, genes controlling the economic traits of fruit trees have been discovered. Some of these traits are fruit skin “anthocyanin defect” in grape [20] and the branch angle (column and weeping type) [21,22], internode length (dwarfing) [23], flesh texture [24], flesh color [25,26,27], and fruit shape (peaches or flat peaches) [28] in peach. In some plants, trichomes are widely distributed in the stem, leaves, fruits, and seeds; they originate from epidermal cells and play a role in protection against biotic and abiotic stressors, with a long evolutionary history, and they protect plants against insect, fungal, and excessive light damage [29,30,31,32]. For instance, the tomato mutant with hairless leaves shows decreased resistance to pest insects [33]. Although in peaches, skin hairiness enhances resistances to biotic and abiotic stress, hairy peaches have greater allergenic potential than nectarines [34].

Recently, several genes related to the development of fruit pubescence have been identified and cloned. For example, Payne et al. found that mutations of *TTG1* and *GL1*, two genes that play a key role in the growth of *Arabidopsis thaliana* hairs, affect the growth of hairs [35]. Li et al. [36] identified Os05g19000 as a candidate gene for the hairiness of the glabrous leaf and hull mutants in rice. Skin hairiness or non-hairiness are commercial characteristics for peach fruit classification. In 2014, Vendramin et al. further narrowed the location range of the key loci of peach/nectarine traits to 1.1 cM and obtained the candidate gene *PpeMYB25*; the authors observed that the insertion of the 7 kb LTR reversal loci in exon 3 of *PpeMYB25* gene was related to the recessive characteristic of nectarines [2], which is in line with our findings. However, no deletion of large fragments in *PpeMYB25* locus was observed. An SNP in ORF of Prupe.5G196400 was found, which is tightly linked to the trait and could be effectively used to distinguish peach and nectarine varieties in germplasm resources.

In this study, we used next-generation sequencing and SNP markers to perform the fine mapping of the gene controlling peach/nectarine traits and obtained the candidate gene *Prupe.5G196400*. Although our gene has not yet been functionally verified, through our 122 peach varieties (lines) and four populations, this marker can distinguish the peach/nectarine characteristics of peaches with 100% accuracy. For this reason, we consider it as a potential candidate gene. Our results provide a foundation for the selection of early follow-up parents, variety identification, early phenotype prediction of progeny representative type, and variety selection in peach breeding.

## 4. Materials and Methods

### 4.1. Plant Material

The BC_1_ lines generated from “Zhongyou No. 4”, the recurrent parent, and “Baihuashanbitao”, the non-recurrent parent, were generated in 2012. “Zhongyou No. 4” is a yellow-flesh and early-ripening nectarine cultivar bred by the Zhengzhou Fruit Research Institute, CAAS, whereas “Baihuashanbitao” is an ornamental peach from *P. davidiana* with female abortion, bred by the Beijing Forestry University.

The parents and 38 BC_1_ individuals were phenotyped as hairy and non-hairy and subsequently genotyped using NGS for mapping. Within the mapped region, SNP markers were designed based on parental sequencing data and tested in the other 129 BC_1_ individuals for fine mapping. To validate the candidate gene, resequencing data for 16 individuals were used, and 122 cultivars or lines were tested to confirm the linkage between genotypes and phenotypes.

### 4.2. DNA Extraction

For the recurrent (Zhongyou No. 4) and non-recurrent (Baihuashanbitao) parents and for the 38 BC_1_ individuals, genomic DNA was extracted using the CTAB method, with slight modifications [37]. In the DNA precipitation step, pure ethanol was added, and the precipitations were picked with a toothpick to acquire high-quality DNA for next-generation sequencing. For the other BC_1_ individuals and 122 cultivars (lines), genomic DNA was isolated with the simplified CTAB method, with one step of chloroform: isoamyl alcohol (*v*/*v*, 24:1) to remove potential contaminants.

### 4.3. Phenotyping

The fruit skin traits (hairiness/non-hairiness) of BC_1_ individuals and the 122 cultivars for mapping and fine mapping were evaluated in the breeding pool (Xinxiang, Henan) by visualization. In addition, the skin was scanned at 30×, using a digital microscope VHX-7000 (Keyence, Osaka City, Japan).

### 4.4. NGS Genotyping and Data Analysis

We used 1.5 μg DNA per sample for DNA resequencing. Sequencing libraries were constructed using the Truseq Nano DNA HT sample preparation kit (Illumina, San Diego, CA, USA) following the manufacturer’s instruction; index codes were added to each sample. Briefly, the DNA sample was sheared by sonication into 350 bp fragments and purified. Each sample was then sequenced using HiSeq 2500 (Illumina, CA, USA), and 150 bp paired-end reads were generated. The peach genome (version 2.0) was used as the reference genome [12].

We applied the BWA (Burrows–Wheeler Aligner) [38] to align the clean reads of each sample against the reference genome (settings: mem -t 4 -k 32 -M -R). Alignment files were converted to BAM files using the SAMtools software 1.6 [39] (settings: -bS -t). Potential PCR duplications were removed using the SAMtools command “rmdup”. If multiple read pairs had identical external coordinates, only the pair with the highest mapping quality remained.

### 4.5. Introgression Line for Mapping

The homozygous SNPs, genotype AA and BB, in two parents were extracted, respectively, from the vcf files for SNPs. Based on the NGS data in the Excel file, a bin map was drawn to identify the introgression fragments from “Baihuashanbitao”. For the mapping analysis, the BC_1_ individuals were divided into two phenotypic classes and then their graphical genotypes were compared in Excel file [40]. We identified chromosomes 1 to 8 to locate the candidate region, in which the genotypes were coincident with the phenotypes.

### 4.6. Primer Design, PCR Amplification, and SNP Genotyping

Based on the resequencing data of parents, the SNPs in the candidate region were selected. We used the Integrative Genomics Viewer 2.3 software [41] to read the BAM file to confirm the SNPs and to design the primers for fine mapping. Primer 3 (http://bioinfo.ut.ee/primer3-0.4.0/ accessed on 18 June 2020) was used to design the primers [42]. For SNP genotyping, the amplified fragment length was approximately 700 bp for Sanger sequencing, which was performed in Sangon Biotech (Shanghai, China); the SNPs were analyzed using the ContigExpress software (2000).

### 4.7. Completely Linked Markers in the Fine-Mapping Region

For the fine-mapped region, the genes were listed referencing the annotation in the GFF file of the peach genome (version 2.0) [43]. Variations in the genes within the fine-mapped region were identified in the BAM files of the two parents in accordance with phenotypes and genotypes, observed in the resequencing data for 16 cultivars, and confirmed in 122 cultivars (lines).

## 5. Conclusions

Using 35 BC_1_ individuals from “Zhongyou No. 4” as the recurrent parent and “Baihuashanbitao” as the non-recurrent parent, the *G* locus was mapped between 14.099 and 16.721 Mb on chromosome 5 and fine-mapped in the range of 481 kb with SNP markers, using the other 129 BC_1_ individuals of this population. A candidate SNP in the gene *Prupe. 5G196400* was obtained based on the resequencing data of other cultivars (lines). The SNP marker was able to identify the phenotypes in 122 cultivars (lines). By genotyping analysis, the SNP was co-segregated with the fruit skin fuzzy phenotype and used for identification of peaches or nectarines in three segregation populations with 100% accuracy.

## Figures and Tables

**Table 1:** Primers used in fine mapping for peach skin hairiness.

Physical Position	Primer Sequence (5’- -3’)		Product Size (bp)	SNP Type
Pp05-SNP-14562747	GGATTAGTGGAGTGGGACAGC	AAGCATCCAGCCAAAACACC	710	G/T
Pp05-SNP-15464780	GAAGAGCAAACTTGGCAGTCC	TTTTGAACCACTTGGGAAGC	763	C/T
Pp05-SNP-15760886	TGACGATTTGAGAGATTGATCG	TTTTTGTGGGAAGAGGAAGG	778	C/T
Pp05-SNP-15858687	ACAGCGTTCGGCTATGAACC	TTTCTTGGGAGTTTTGTGTGC	866	A/G
Pp05-SNP-15959172	CTCTTACGACCAAGAACCAACC	GTTGGTGGAGTGGGAGAAGC	815	C/T
Pp05-SNP-16242580	ACTGGTGGTTTGTTGGTTGG	TTTCCATACATGTCTTAAAGGTTC	633	C/G
Pp05-SNP-16563677	ACGCAATTGGCAGTTACACC	CCACCAGAGGCTAGAGTTGC	600	T/C

**Table 2:** Genotyping results for 16 cultivars (lines) regarding skin hairiness.

Cultivars (Lines)	Phenotype	Genotype
Zhongyou No. 4	Non-hairy	T/T
Baihuashanbitao	Hairy	G/G
10–7	Hairy	T/G
96–51	Hairy	T/G
Bairuyu	Hairy	G/G
ludong-2-04	Non-hairy	T/T
P7-12-03	Non-hairy	T/T
Shanza-02	Hairy	T/G
Shuipingzhi	Hairy	T/G
Weni2	Hairy	G/G
yb144	Hairy	T/G
Zhongpan No. 01	Hairy	T/G
Zhongtao No. 05	Hairy	T/G
Zhongyou No. 08	Non-hairy	T/T
Zhongyou No. 13	Non-hairy	T/T
Zhongyou No. 20	Non-hairy	T/T

**Table 3:** The SNP marker was used for MAS in three segregation populations to predict the phenotype with 100% accuracy.

Segregation Populations		Individuals	Hairy	Non-Hairy	Accuracy
Zhongyou No. 8 (non-hairy)	Beijingduan (hairy)	30	13	17	100%
09-bei8-25 (hairy)	Zhongtaobaiyu (hairy)	196	196	0	100%
Zhongtao No. 5 (hairy)	Zhongyou No. 15 (non-hairy)	15	0	15	100%

## Supplementary Materials

The following are available online at https://www.mdpi.com/article/10.3390/plants10071433/s1. Figure S1: The IGV snapshot of SNP mutation in gene Prupe.5G196400 based on resequencing data of two parents, Figure S2: The IGV snapshot of SNP for 16 cultivars (lines) in candidate gene Prupe.5G196400, Data S1: Complete linkage region with fruit skin hairiness/ non-hairiness based on the genotype and phenotype of 35 BC1 introgression individuals and two parents, Data S2: The genotype of 129 BC1 individuals, ten recombinants and two parents used for fine mapping. The fruit skin was labelled with hairiness and non-hairiness, Data S3: The genes listed in the mapped region, containing 86 genes, Data S4: The SNP marker was used to validate the genotype and phenotype in 122 cultivars (lines).
