# Supplementary figures and images for: Fine Mapping of the Gene Controlling the Fruit Skin Hairiness of Prunus persica and Its Uses for MAS in Progenies
Source: Plants (Basel). 2021 Jul 14;10(7):1433. doi: 10.3390/plants10071433 (PMC8309289; doi:10.3390/plants10071433)

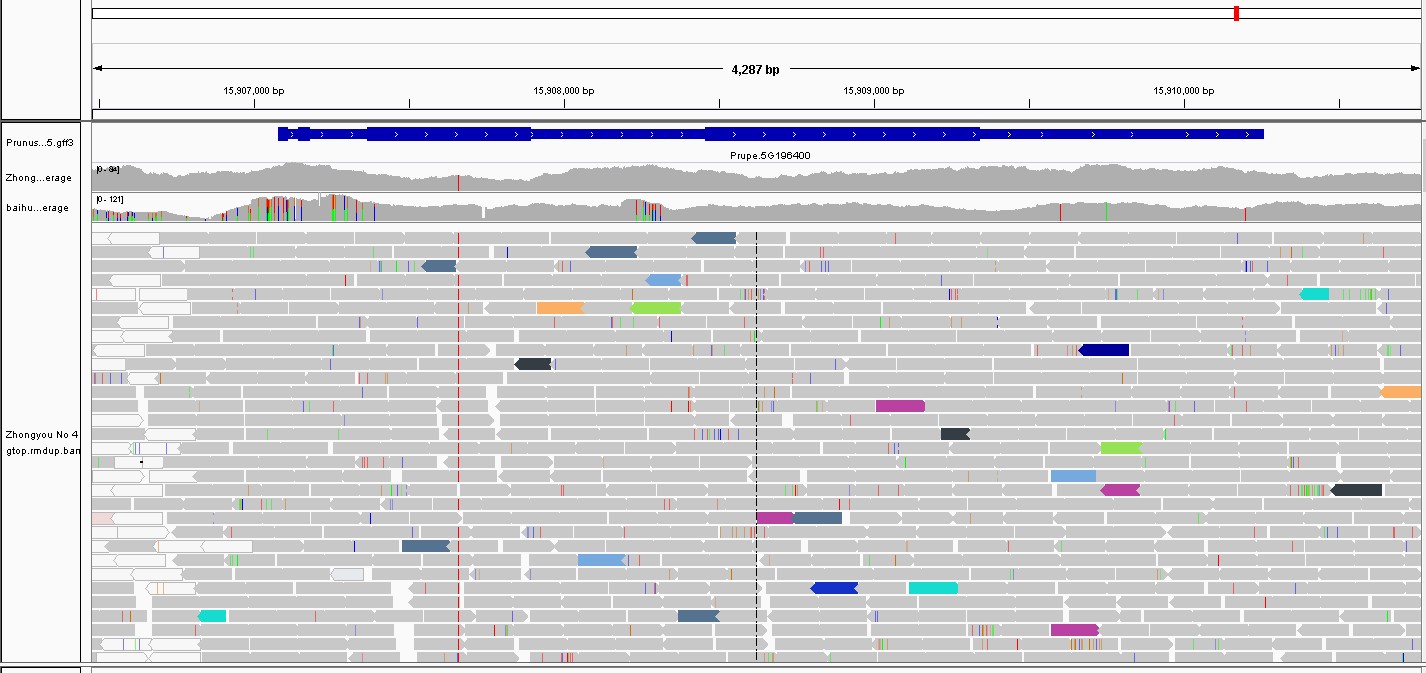

Supplement: Supplementary file 1 [file plants-10-01433-s001.zip › plants-1168794-supplementary/Supplementary figure 1.jpg]

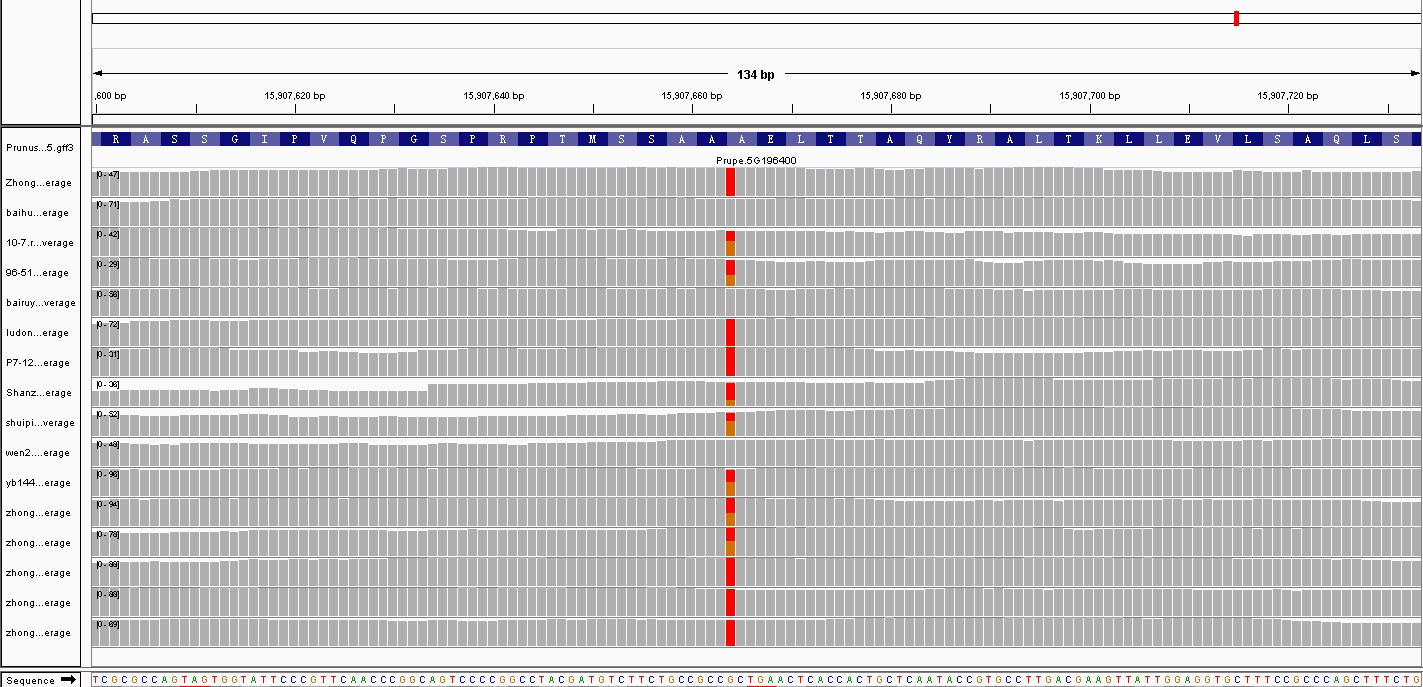

Supplement: Supplementary file 1 [file plants-10-01433-s001.zip › plants-1168794-supplementary/Supplementary figure 2.jpg]
